# Less than half of the European dietary recommendations for fish consumption are satisfied by national seafood supplies

**DOI:** 10.1007/s00394-021-02580-6

**Published:** 2021-05-17

**Authors:** Anneli Lofstedt, Baukje de Roos, Paul G. Fernandes

**Affiliations:** 1 School of Biological Sciences, University of Aberdeen, Aberdeen, AB24 2TZ UK; 2 The Rowett Institute, University of Aberdeen, Foresterhill, Aberdeen, AB25 2ZD UK

**Keywords:** Seafood, Net supply, Dietary recommendations, Europe

## Abstract

**Purpose:**

To review the seafood dietary recommendations of European countries and compare them to national seafood supplies.

**Methods:**

Current seafood dietary recommendations were collated from national health authorities across Europe. Food balance sheets were downloaded from the FAO, and appropriate conversion factors were applied to each seafood commodity. Average net per capita seafood supplies from 2007 to 2017 were derived from data on imports and production for food from both capture fisheries and aquaculture, accounting for exports.

**Results:**

Both national dietary recommendations and seafood supplies varied considerably throughout Europe. At a national level, on a per capita basis, only 13 out of the 31 of European dietary recommendations for fish consumption were satisfied by national seafood supplies. Most of the countries with coastal access, as well as those with traditional fish-eating cultures, such as France and countries in Northern Europe, had adequate seafood supplies to meet their recommendations. The landlocked countries of Central and Eastern Europe did not have enough seafood supplies to satisfy their recommendations.

**Conclusions:**

Our findings emphasise the need to not only consider consumer health outcomes when developing and advocating dietary recommendations, but also the sustainability of food production systems. As many foods are not necessarily locally sourced but traded as part of global production and distribution systems, it is important to consider greater consistency between national dietary recommendations to facilitate more sustainable marine food systems.

**Supplementary Information:**

The online version contains supplementary material available at 10.1007/s00394-021-02580-6.

## Introduction

The global fish production industry plays an important role in national economies, supporting an estimated 59.5 million jobs in the primary sector of capture fisheries and aquaculture [1]. Seafood is also the most valuable traded food commodity worldwide, with net exports from developing countries valued at $38 billion in 2018, exceeding that of sugar, tobacco, meat and rice combined [1]. Fish and fisheries products also play a vital role in global food security, particularly in developing countries, providing around 17% of animal protein consumed by the global population in 2017 [1]. In developing countries, fish offers a cheap source of high-quality protein and diversity to a diet dominated by more staple foods such as maize and rice [2]. In these countries, fish is also a valuable contributor to the reference nutrient intakes for a range of micronutrients and, therefore, fish consumption may contribute to alleviating highly prevalent micronutrient deficiencies [3]. A number of studies have highlighted the contribution of fish consumption to adequate intakes of micronutrients on a global scale [4–6]. A recent modelling approach linking nutrient availability from marine fish to nutrient deficiencies in 43 countries found that nutrients available in marine finfish exceed that of dietary requirements, but only for populations residing within 100 km of the coast [6]. Consumption of seafood is also linked to health benefits, such as a reduction in the risk of mortality of coronary heart disease [7]. Compared with very low fish intake (i.e., < 1 serving/month), low fish intake (1 serving/week) reduces the risk for coronary heart disease and stroke by 16% and 14%, respectively, and moderate fish intake (2–4 servings/week) reduces risk for coronary heart disease and stroke by 21% and 9%, respectively [8, 9].

Given these nutritional and health benefits, many countries have established recommendations for seafood consumption as part of their national dietary recommendations. Some of these recommendations are based on cohort studies that focus on total seafood consumption, while others are based on the content of the main omega-3 fatty acids eicosapentaenoic acid (EPA) and docosahexaenoic acid (DHA) [7, 10]. Moreover, fish are an important source of bioavailable micronutrients, often lacking in plant-based diets [11, 12] and enhance the availability of minerals from cereal-based foods [13]. They also offer an alternative, more-affordable animal-based product [13] with a lower environmental impact [14]. Few countries take the environmental perspective into account in their recommendations for fish consumption [10]. Such an environmental perspective is important due to increasing constraints on the global seafood supply from growing populations, growing disposable incomes, and therefore, increased demand [1]. For example, in a previous study of the UK, fish supply only satisfied 64% of the amount proposed by dietary recommendations [15]. Therefore, national aspirations for increased fish consumption may have wider environmental implications.

Marine capture fisheries represent a large proportion of total fish production, but production from this sector has remained largely stagnant for the past three decades. Broad concern about the overexploitation of wild fish stocks and environmental impacts of aquaculture have been raised over the years. At the start of the twenty-first century, declines in fish stocks were widely reported [16, 17], but stringent management measures have been effective at rebuilding many assessed stocks to above sustainable target levels at European and global levels [18, 19]. However, the majority of global fish stocks are unassessed and, therefore, their status is uncertain [19]. Aquaculture currently provides around half (46%) of global fish production [1], but concerns have been raised about the nutritional quality of farmed fish compared to wild varieties, especially in relation to the former’s lower content of omega-3 fatty acids and some micronutrients [3, 10].

This paper provides an analysis of seafood supplies, defined as the amount of seafood available for human consumption, by examining fish production (accounting for imports and exports) from both wild capture fisheries and aquaculture. Dietary recommendations of European countries were reviewed to determine if they were satisfied by seafood supplies at the national level. Such information is important considering the implications for dietary recommendations and human health, as well as a sustainable supply of seafood products.

## Methods

### Dietary recommendations in European countries

In the past decades, countries have developed unique food-based dietary recommendations based on country-specific intakes of nutrients, and cultural trends. Given the diversity of fish consumption levels between European countries, a general recommendation for fish consumption is not provided for Europe [20]. Some countries provide additional separate recommendations for different demographic groups such as infants, pre-school children, adolescents and pregnant women. Albania, for example, provides eight dietary recommendations according to age class: new-borns, 1–2 years old, 2–3 years old, 4–6 years old, 6–12 years old, 13–18 years old, adults and the elderly. However, not all countries provide such detail, thus only one recommendation for adults or the general population was considered. Nevertheless, where recommendations were provided for children, they were set to be half the adult recommendation, which we considered when calculating net seafood supplies.

Current national dietary recommendations for seafood (finfish and shellfish) for adults in Europe were sourced from national public health authorities and translated. Of the 40 European countries examined (i.e., those countries with corresponding production and trade data), only 31 countries were found to have quantified fish-based dietary recommendations. Five countries (Belarus, Moldova, Montenegro, Russia and Serbia) did not have recommendations for fish consumption and four countries (Luxembourg, North Macedonia, Portugal and Slovakia) did not recommend weekly consumption frequency. For example, North Macedonia’s dietary recommendation is to “substitute meat and meat products with fish, poultry and beans” and Portugal’s recommendation is to “eat 1.5 to 4.5 portions of fish, meat, eggs per day”.

For each dietary recommendation, portion size and consumption frequency per week were considered. If portion size was not quantified, it was assumed one portion size was equal to 100 g [15]. When a range of portion sizes were provided (such as in Bulgaria), the mean of the range was taken.

### European fish production and trade

Food balance sheets of fish and fisheries products between 2007 and 2017 were downloaded from the Food and Agricultural Organisation (FAO) Fishery and Aquaculture Statistical Time series (released in September 2020) (FishStatJ, version 4.00.16.) [21]. Compared to other FAO data sources, fishery products in the food balance sheets do not represent individual commodities, but the species are aggregated into eight main groups of similar characteristics reflecting the International Standard Statistical Classification of Aquatic Animals and Plants (ISSCAAP) classification (Supplementary material, Table 1). This includes wild capture and aquaculture statistics for all finfish, crustaceans and molluscs from marine, freshwater and brackish environments. Aquatic animals (such sea cucumbers, turtles and sea urchins) were omitted as they are not included in dietary recommendations for seafood consumption. For the purpose of this study, the term “seafood” refers to marine, brackish and freshwater finfish, shellfish, cephalopods and molluscs.

**Table 1:** Current national dietary recommendations for adults (serving per week and portion size) in 31 European countries

Country	Organisation^a^	National recommendations (portions/servings of fish per week)^b^	Portion/serving size (g)	Mean recommended intake (g/week)
Albania	Albanian Ministry of Health	2 or 3	100–120	275
Austria	Austrian Ministry for Health	1–2	150	225
Belgium	Federal Public Service Health, Food Chain Safety and Environment	1–2 (one of which should be oily)	100	150
Bosnia and Herzegovina	Bosnian Institute for Public Health	1	100^c^	100
Bulgaria	Bulgarian Ministry of Health	1–2	150–200	263
Croatia	Croatian Ministry of Health	1–2	100^c^	150
Czech Republic	Czech Society for Nutrition	400 g	^d^	400
Denmark	Danish Ministry of Food, Agriculture and Fisheries	350 g	^d^	350
Estonia	Estonia National institute for Health Development	3	75	225
Faroe Islands	Danish Ministry of Food, Agriculture and Fisheries	350 g	^d^	350
Finland	Finish National Nutrition Council	2–3	100–150	313
France	French Ministry of Health	2	100	200
Germany	German Nutrition Society	1 or 2	100^c^	150
Greece	Greek Institute for Preventative Environmental and Occupational Medicine	2 or 3	150	375
Hungary	National Institute for Food and Nutrition Science	1	150	150
Iceland	Icelandic Directorate of Health	2–3 (one of which should be oily)	150	375
Ireland	Irish Department of Health	2 (both oily)	100^c^	200
Italy	CREA Food and Nutrition Research Centre	2–3	100	250
Latvia	Latvian Ministry of Health	2	100–140	240
Lithuania	Lithuanian Ministry of Health	2–3	100^c^	250
Malta	Health Promotion and Disease Prevention Directorate	2 (one of which should be oily)	115	230
The Netherlands	Dutch Health Council	1 (one of which should be oily)	100	100
Norway	Norwegian Nutrition Council	2 or 3 (~ 300 to 450 g)	^d^	375
Poland	Polish National Institute of Public Health	2 (one of which should be oily)	100^c^	200
Romania	National Food and Nutrition Committee	2 or 3	100^c^	250
Slovenia	Slovenian National Institute of Public Health	2	100^c^	200
Spain	Spanish Agency for Food and Nutrition Safety	3–4	125–150	482
Sweden	Swedish Food Agency	2–3 (one of which should be oily)	100^c^	250
Switzerland	Swiss Society of Nutrition	1–2	100–120	165
Ukraine	Ukrainian Ministry of Public Health	20 g of fish per day	20	140
United Kingdom	Scientific Advisory Committee on Nutrition	2 (one of which should be oily)	140	280

^a^Information retrieved from: Albania [http://www.fao.org/3/a-as658e.pdf (2008)]; Austria [https://www.sozialministerium.at/Themen/Gesundheit/Lebensmittel-Ernaehrung/Ern%C3%A4hrungsempfehlungen/Ern%C3%A4hrungspyramide0.html (2019)]; Belgium [https://www.nice-info.be/voedingsmiddelen/nieuwe-voedingsaanbevelingen-focussen-op-voedingsmiddelen (2019)]; Bosnia and Herzegovina [http://www.fao.org/3/a-as669o.pdf (2004)]; Bulgaria [http://ncpha.government.bg/files/hranene-en.pdf (2006)]; Croatia [http://www.udruga-hzn.com/uploads/4/8/2/9/48294743/nacionalne_smjernice_za_prehranu_ucenika_u_osnovnim_skolama.pdf (2013)]; Czechia [http://www.vyzivaspol.cz/vyzivova-doporuceni-pro-obyvatelstvo-ceske-republiky/ (2012)]; Denmark [https://altomkost.dk/materialer/publikation/pub/hent-fil/publication/de-officielle-kostraad/ (2015)]; Estonia [https://toitumine.ee/kuidas-tervislikult-toituda/toidusoovitused/kala-linnuliha-liha-ja-muna (2015)]; Faroe Islands [https://altomkost.dk/materialer/publikation/pub/hent-fil/publication/de-officielle-kostraad/ (2015)]; Finland [https://www.ruokavirasto.fi/en/themes/healthy-diet/nutrition-and-food-recommendations/adults/ (2019)]; France [https://www.mangerbouger.fr/Les-recommandations/Aller-vers/Le-poisson (2019)]; Germany [https://www.dge.de/ernaehrungspraxis/vollwertige-ernaehrung/10-regeln-der-dge/ (2017)]; Greece [http://www.diatrofikoiodigoi.gr/files/PDF/ADULTS.pdf (2014)]; Hungary [http://mdosz.hu/hun/wp-content/uploads/2016/10/mdosz_kreativ_v25_eng.pdf) (year unknown)]; Iceland [https://www.landlaeknir.is/servlet/file/store93/item25796/R%C3%A1%C3%B0leggingar%20um%20matar%C3%A6%C3%B0i%20LR_20.01.2015.pdf (2017)]; Ireland [https://www.gov.ie/en/publication/da7f19-eat-well/# (2019)]; Italy [https://www.crea.gov.it/documents/59764/0/LINEE-GUIDA+DEFINITIVO+%281%29.pdf/3c13ff3d-74dc-88d7-0985-4678aec18537?t=1579191262173 (2018)]; Latvia [https://esparveselibu.lv/sites/default/files/inline-files/VM_Uztura_ieteik_pieaug.pdf (2020)]; Lithuania [http://www.smlpc.lt/media/file/Skyriu_info/Metodine_medziaga/Sveikos_mitybos_rekomendacijos_2010.pdf (2010)]; Malta [https://deputyprimeminister.gov.mt//en/health-promotion/documents/library/publications/healthy%20plate%20en.pdf (2015)]; the Netherlands [https://www.voedingscentrum.nl/nl/gezond-eten-met-de-schijf-van-vijf/wat-staat-er-in-de-vakken-van-de-schijf-van-vijf/vis-peulvruchten-vlees-ei-noten-en-zuivel.aspx (2015)]; Norway [https://www.helsedirektoratet.no/faglige-rad/kostradene-og-naeringsstoffer (2016)]; Poland [https://ncez.pl/upload/talerz-i-zalecenia.pdf (2020)]; Romania [https://www.spitalsmeeni.ro/docs/ghiduri/ghid_alimentatie_populatie.pdf (2006)]; Slovenia [https://www.nijz.si/sites/www.nijz.si/files/publikacije-datoteke/12_korakov_plakat_0.pdf (2018)]; Spain [https://www.aesan.gob.es/AECOSAN/docs/documentos/nutricion/alimentacion_sana_para_todos.pdf (2010)]; Sweden [https://www.livsmedelsverket.se/globalassets/publikationsdatabas/andra-sprak/kostraden/kostrad-eng.pdf (2015)]; Switzerland [https://www.sge-ssn.ch/fr/fragenkatalog/substances-nutritives/ (2017)]; Ukraine [https://www.euro.who.int/__data/assets/pdf_file/0017/150083/E79832.pdf (2003)]; United Kingdom [https://assets.publishing.service.gov.uk/government/uploads/system/uploads/attachment_data/file/618167/government_dietary_recommendations.pdf (2016)]

^b^Portions or servings of fish per week, unless otherwise stated. Recommendations for dependencies and other territories were the same as their hosts

^c^Where portion size was not provided, it was assumed to equal to 100 g

^d^Weekly dietary recommendation without mention of a portion size

In the food balance sheets, species aggregations are measured as live weight equivalent in tonnes without accounting for processing losses, over-representing the amount available for human consumption. To account for this, the elements were separated into finfish and shellfish and landed weight was converted to processed weight, using conversion weight ratios provided by HM Revenue and Customs (HMRC), as used previously [15]. The average conversion factor from whole fish to fillets equated to edible proportions by weight of 0.49 (SE ± 0.02) for finfish and 0.28 (SE ± 0.05) for shellfish, cephalopods and molluscs. Commodities were then separated into their pre-defined elements; imports, production, non-food uses and exports. Non-food uses included aquatic products destined for fish meal and oil, feed and bait, ornamental purposes and additional non-food uses for fish production (such as fertilisers and medical uses).

### National population size in Europe

Estimates of population size between 2007 and 2017, for adults (15–64 years), the elderly (greater than 65 years old) and children (less than 15 years old) were obtained from the World Bank [22] to express the data in per capita terms. Cohort population data for the Faroe Islands in 2017 were sourced from Statista [23]. Annual cohort data for the Faroe Islands were not readily available, thus we acknowledge seafood per capita supply may be inaccurate for this country.

### Calculation of net seafood supplies for human consumption (g/capita/week)

Net seafood supplies for human consumption per capita were estimated per week (g/capita/week) at the national level (Eq. 1). This represents seafood production less non-food uses, plus associated imports, after accounting for exports (modified from [21]). Thus, net seafood supply (in grams) per country, per capita, per week, was estimated as

1 $${\text{Net}}\,{\text{seafood}}\,{\text{supply}} = \left( {\left( {{\text{production}} - {\text{non-food}}\,{\text{uses}}} \right) + {\text{imports}}} \right) - \left( {{\text{exports}}} \right)/{\text{population}}\,{\text{size}}/52.$$

Net seafood supply at a national level was calculated on an annual basis between 2007 and 2017 to assess inter-annual variation. Comparisons between average net seafood supply data (between 2007 and 2017) and national dietary recommendations were subsequently made. Net seafood supply in the Faroe Islands dropped below 0 g/capita/week in 2010 thus this data point was removed.

## Results

### National dietary recommendations

Current adult fish consumption recommendations varied considerably throughout Europe with recommendations ranging from 100 to 482 g/capita/week (Table 1). Most countries recommend a minimum serving of two portions of fish per week, equivalent to 150-300 g/capita/week, depending on portion size, which varied between 20 g and up to 175 g. Weekly dietary recommendations were highest in Spain (482 g) and the Czech Republic (400 g). Greece, Iceland and Norway all recommended 375 g/capita/week. Dietary recommendations were lowest in Bosnia and Herzegovina and in the Netherlands, recommending a weekly fish intake of just 100 g/capita/week.

### Inter-annual variability in net national seafood supplies between 2007 and 2017

**Fig. 1:**
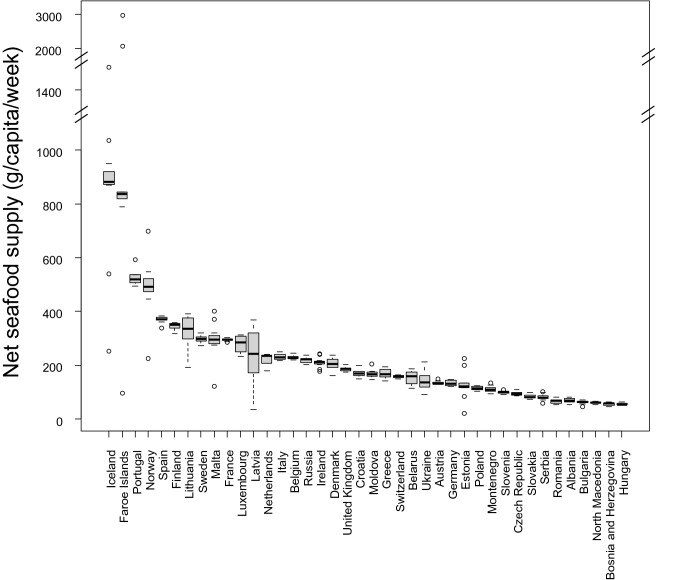
Boxplot of annual variability in seafood supplies to European countries between 2007 and 2017. The boxplot represents the spread of seafood supplies over the 10-year period; the solid horizontal line in the boxplot represents the median; the hinges i.e., border ends of the boxes represent the 25% and 75% quartiles; the lines, or “whiskers” from the hinges represent 95% confidence intervals; the hollow dots beyond the extremes of the whiskers represent outliers

**Fig. 2:**
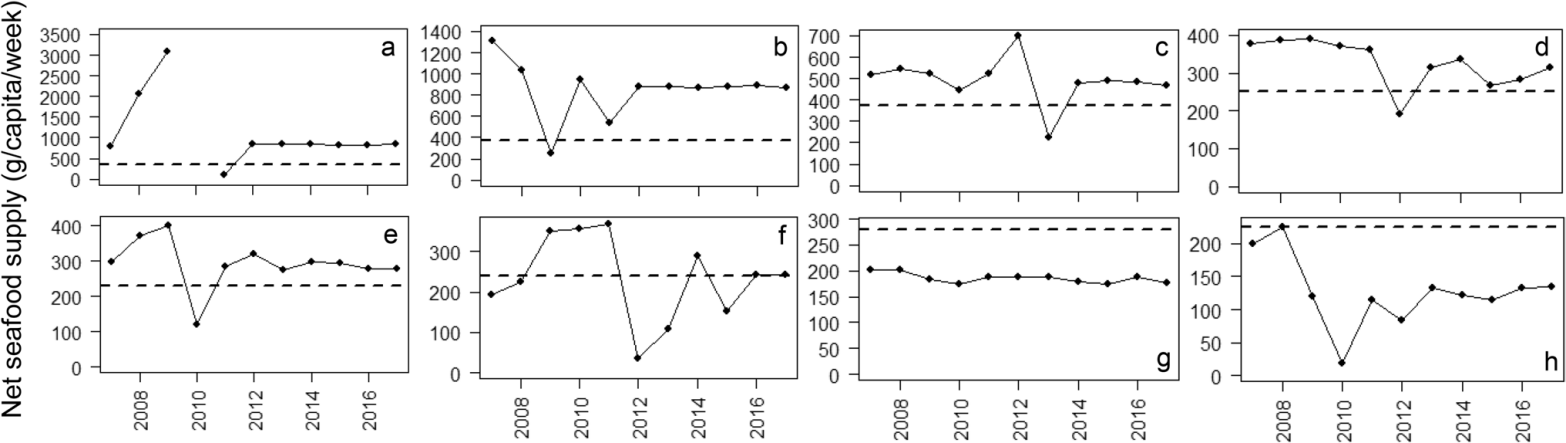
Annual net seafood supplies for human consumption between 2007 and 2017 in selected countries identified to have a higher than average variation in seafood supply between years (except UK); **a** Faroe Islands, **b** Iceland, **c** Norway, **d** Lithuania, **e** Malta, **f** Latvia, **g** UK, **h** Estonia. The dashed lines represent national dietary recommendations for adults

Median net seafood supplies were greatest not only in Northern Europe (Iceland, Faroe Islands, Norway, Finland) but also in Portugal and Spain. Net seafood supplies were lowest in Hungary, Bosnia and Herzegovina and North Macedonia (Fig. 1). Generally, net national seafood supplies were constant between 2007 and 2017 across the 40 countries. However, seven countries showed some year-to-year variability in net seafood supply over this 10-year period (Fig. 2). Between 2007 and 2017, Faroe Islands and Iceland showed the greatest range of seafood supplies (20-3084 g/capita/week and 252-1310 g/capita/week, respectively). Such inter-annual analysis highlights that in some years, dietary recommendations were not satisfied. No obvious common inter-annual trends were observed in seafood supplies between countries.

### Average net national seafood supplies between 2007 and 2017 versus national dietary recommendations for fish consumption in adults

**Fig. 3:**
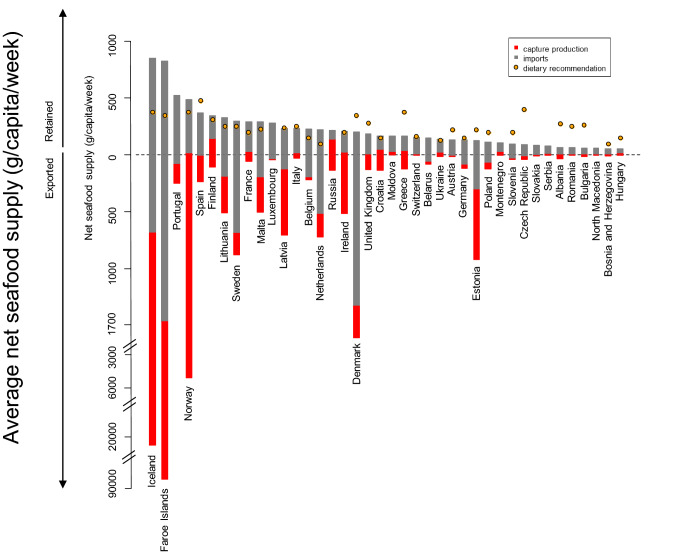
Average net seafood supplies for human consumption in Europe (g/capita/week), between 2007 and 2017, relative to dietary recommendations for adults. The total length of both bars for each country indicates the total amount of seafood supply, accounting for national production, imports and exports. Red bars indicate national production and grey bars indicate imports. The position of the bars for each nation on the *y*-axis indicates the net supply to the nation: quantities above the dotted line (*y* = 0) indicate the amount retained for national consumption; quantities below the dotted line (*y* = 0) indicate the amount exported (and re-exported, as seen in Denmark and Iceland for example). Orange dots indicate the national adult recommendations for fish consumption. Seafood supplies for European countries which lack fish dietary recommendations and those which are not quantified, are also included

Average net national seafood supplies between 2007 and 2017, in relation to national dietary recommendations for fish consumption in adults, varied throughout Europe (Fig. 3). Net seafood supply ranged from 55 to 851 g/capita/week, with an average of 224 g/capita/week.

Seafood production was highest in Northern Europe (Faroe Islands, Iceland, Norway and Denmark), with Estonia, Latvia and Ireland also recording high seafood production (> 600 g/capita/week). Luxembourg, Austria and Switzerland recorded the lowest seafood production (> 5 g/capita/week). Seafood imports were greatest in the Faroe Islands, Denmark and Iceland (> 1000 g/capita/week), whilst, Hungry, Bosnia and Herzegovina, North Macedonia and Bulgaria imported the least amount of seafood in Europe (< 56 g/capita/week). Seafood exports were also greatest in Northern Europe (Faroe Islands, Iceland, Norway, and Denmark), exceeding 1000 g/capita/week. Moldova and Switzerland exported less than 1 g/capita/week. The majority of European countries exported previously imported seafood, with the Faroe Islands, Iceland and Denmark being the largest re-exporters.

Our results indicated that on average, at a national level, only 13 of the 31 dietary recommendations for fish consumption in Europe were satisfied by net seafood supplies. Many Central and Eastern European countries were unable to satisfy their dietary recommendations, mostly attributed to low national production and higher than average dietary recommendations. Our findings also suggest that if imports ceased and fish commodities were still exported, recommendations would not be satisfied by national production in any of the European countries examined. However, should trade (i.e., imports and exports) cease and countries retain their capture production, then 10 of 31 countries’ recommendations would be satisfied: Iceland, Faroe Islands, Norway, Lithuania, Latvia, Netherlands, Ireland, Denmark, Croatia and Estonia.

## Discussion

We found that national dietary recommendations across Europe varied significantly between countries, ranging from 100 to 482 g/capita/week. Between 2007 and 2017, national dietary recommendations for fish consumption were only satisfied by net seafood supplies in 13 out of 31 European countries. Notably, these countries all have large coastal access or traditional fish-eating cultures. Net seafood supplies were lowest in landlocked countries and those with low production and import rates.

Many European dietary recommendations for fish consumption are underpinned by evidence that fish consumption is associated with a reduced risk of mortality of coronary heart disease [10]. The beneficial effects of fish consumption have historically been attributed to its content of omega-3 fatty acids [7]. A recent systematic assessment of the effects of these fatty acids, mostly provided as fixed-dose supplements, on cardiovascular health outcomes, indicated that increasing consumption had little or no effect on mortality or cardiovascular health [24]. However, the health benefits of fish consumption on cardiovascular health outcomes are well established [8, 9] and may be greater than the sum of its individual constituents such as omega-3 fatty acids [10]. Fish is an important source of protein, long chain *n*-3 polyunsaturated fatty acids (LC *n*-3 PUFA), vitamins and minerals [10], and the importance of fish consumption for nutritional status appears to be of high significance, especially in low- and middle-income countries [4, 5, 10]. It has been hypothesised that global net supply of mainly wild catch fish could significantly impact micronutrient deficiencies in the future, especially for coastal regions [5]. However, increasingly, demand for fish is being met by aquaculture production [1] and its nutritional quality has been questioned. The marine finfish aquaculture industry has increasingly sourced fish feed from terrestrial agriculture to become more cost effective. The introduction of vegetable oils and meals to fish feeds has affected the nutritional composition of farmed fish, resulting in lower levels of omega-3 fatty acids and micronutrients over the past decade [10, 25]. Therefore, we may need to eat more fish to provide similar health benefits than those described previously [8, 9]. Existing dietary recommendations for fish intake are based on cohort studies that were performed with mostly capture fish that probably had higher levels of omega-3 fatty acids and micronutrients, and future recommendations will need to take account of how this might change.

The literature is not very clear on the positioning of seafood within a sustainable diet. Indeed, seafood consumption is commonly presented as a dilemma. The trade-offs between the health benefits of eating seafood, the lower impact of fish consumption on greenhouse gas emissions compared with other animal-based protein such as beef and pork, and concerns of overfishing and ecological impacts are not well defined. Furthermore, farmed and wild‐capture production methods are often not integrated into research on the impacts of diets and future food scenarios [26]. To position seafood within a sustainable diet, it can be argued that greater consideration needs to be given to fish supply when considering dietary recommendations for fish consumption. Our findings for the UK, i.e., that dietary recommendations are not satisfied by net fish supply, agree with those reported by Thurstan and Roberts [15], who concluded that recommended levels of fish consumption were not achievable by net supply in 2012. They found that total supplies of fish in that year only met 64% of recommended fish intake. Our average results from data obtained between 2007 and 2017 were similar: fish supply met 66% of recommended dietary fish intake (185 g of the recommended 280 g was available). A comparison of global fish consumption with regional fish supplies to determine which areas meet demand by production and/or imports using population and catch data for 64 Large Marine Ecosystems (LME’s) found that two-thirds of LME’s reported landings were not sufficient to meet local consumption [27].

Between 2007 and 2017, net seafood supplies were relatively consistent in most European countries. However, there was more variation in net seafood supplies across years in some countries, including those with higher levels of fish production (Faroe Islands, Iceland, Norway, Lithuania, Malta), but also in those with lower levels of fish production (such as Estonia) (Fig. 2). Seafood supply may vary annually due to changes in fish stocks status, consumer demand and socio-economic factors. Thurstan and Roberts [15] also reported annual variability in fish supply in the UK and noted that fish supplies met the recommended level of intake for fish only twice in 124 years.

We appreciate the term “seafood” can be misleading when describing fish supplies in landlocked countries, which have no marine fisheries. Whilst, inland freshwater fisheries contribute little to European fish production, Czech Republic, Hungary and Austria rely on freshwater aquaculture and capture production. Although in this analysis, “seafood” consisted of finfish, shellfish, molluscs and cephalopods from brackish, marine and freshwater systems, we propose that “aquatic protein” or “aquatic food” could be used in the future. “Aquatic food” would also include seaweed, the consumption of which has been postulated to have been significant in the past [28] and may increase in future [29], but is not considered here.

Within the past decade, trade of wild and farmed fishery products has contributed globally to economic growth and food security. Whilst fisheries contribute little to the GDP and food security in developed countries, in Iceland and the Faroe Islands, fish is vital to the national economy [30], with fish exports exceeding 40% of the total value of merchandise traded [1]. In our analysis, we assumed that countries prioritised exporting national fish production over imported fish. Although our results suggest that some imported seafood commodities are re-exported, after accounting for all exports no national dietary recommendations are satisfied by national production alone if exports continued. With limited growth in capture production, the EU is increasingly relying on extra-EU imports to meet demand [1]. Moreover, a recent WWF analysis predicted that many people living in poverty will choose to export fish rather that consume it by 2050 [27]. Therefore, to assure a sustainable supply of fish for current and future generations, greater consistency in national dietary recommendations would aid in the development of more sustainable food systems.

Marine model projections predict climate-induced shifts in fish distributions which will decrease the maximum catch potential by 2050, thereby threatening global supply for human consumption [30]. For example, changes to Atlantic mackerel migration has already led to a breakdown of international management agreements [31]. Additional factors such as habitat degradation and pollution will also affect fish abundance, with a recent report stating a 76% decline in global migratory freshwater fish populations over the last 50 years [32]. There are alternative sources of omega-3 fatty acids supplied into the food system through an expanding aquaculture sector [33], and through the development of new sources of EPA and DHA, such as algal biomass and GM oils, especially for the production of aquafeeds [34]. An alternative plant-based dietary source of omega-3 fatty acids, alpha linolenic acid (ALA), is, however, not found to produce the same health benefits as the marine-derived omega-3 fatty acids EPA and DHA, and the conversion of ALA to EPA and DHA is limited in humans [35].

Strengths of this study include linking dietary recommendations with net seafood supplies with a European perspective, using the most recent food balance sheets from the FAO, providing a comprehensive picture of a country’s food seafood supply and allowing the tracking of fish supply patterns over time. However, care should be taken when comparing production, imports and exports between countries. Calculating fish supplies in g/capita/week may introduce a bias towards those countries with smaller population sizes. For example, over the same 10-year period, Norway, the Netherlands and Spain exported the highest amount of seafood (in grams) in Europe. However, Iceland and Faroe Islands recorded higher exports (g/capita/week) owing to the significantly smaller population sizes of these countries. Also, in our analysis, seafood supplies may be underestimated, especially in southern Europe due to under-reporting of subsistence fisheries. Small-scale fisheries represent 84% of the total fishing fleet by number of vessels in some southern European regions, particularly around the Mediterranean and the Black Sea [36]. On the other hand, supplies are likely to be over-estimated across European countries as food balance sheets do not account for food waste at the retail or household level. Furthermore, it should be noted that the majority of European recommendations for fish consumption are for finfish only and do not include other seafood products such as crustaceans and molluscs; commodities which were included in our calculations of national seafood supply.

In conclusion, our findings emphasise the need to not only consider consumer health outcomes when developing and advocating dietary recommendations, but also seafood supplies and the sustainability of food production systems. As many foods are not necessarily locally sourced, but part of global production and distribution systems, it is important to consider a greater consistency between national dietary recommendations to aid the development of more sustainable marine food systems.

## Supplementary Information

Below is the link to the electronic supplementary material.

Supplementary file1 (DOCX 14 KB)

## Data Availability

The datasets presented in this study were sourced from the fishery food balance sheets workspace using Software for Fishery and Aquaculture Statistical Time Series (FishStatJ) (http://www.fao.org/fishery/statistics/software/fishstatj/en).
