# Supplementary material for: Less than half of the European dietary recommendations for fish consumption are satisfied by national seafood supplies
Source: Eur J Nutr. 2021 May 17;60(8):4219–28. doi: 10.1007/s00394-021-02580-6 (PMC8572203; doi:10.1007/s00394-021-02580-6)
Supplement: Supplementary file 1 — Supplementary file1 (DOCX 14 KB) [file 394_2021_2580_MOESM1_ESM.docx]

**SUPPLEMENTARY MATERIAL**

**Table 1.** Eight groups of aggregated species, as defined by the International Standard Statistical Classification of Aquatic Animals and Plants (ISSCAAP).

| ISSCAAP group | Examples |
| --- | --- |
| Freshwater and Diadromous fish | including carps, barbels, tilapias, sturgeons, eels, salmons, trouts, shads, etc |
| Demersal fish | including flatfishes, cods, hakes, haddocks, redfishes, sharks, etc. |
| Pelagic fish | including anchovies, herrings, sardines, tunas, mackerels, etc. |
| Marine fish, other | including unidentified marine fish. |
| Crustaceans | including crabs, lobsters, shrimps, krill, etc |
| Molluscs excl. Cephalopods | including abalones, oysters, mussels, scallops, clams, etc. |
| Cephalopods | including squids, cuttlefishes, octopuses, etc. |
| Aquatic animals, others | including frogs, turtles, sea-cucumbers, sea-urchins, etc |
